# Oral arsenic administration to *humanizedUDP-glucuronosyltransferase1* neonatal mice induces UGT1A1 through a dependence on Nrf2 and PXR

**DOI:** 10.1016/j.jbc.2023.102955

**Published:** 2023-01-30

**Authors:** Xiaojing Yang, André A. Weber, Elvira Mennillo, Miles Paszek, Samantha Wong, Sabrina Le, Jia Ying Ashley Teo, Max Chang, Christopher W. Benner, Robert H. Tukey, Shujuan Chen

**Affiliations:** 1 Laboratory of Environmental Toxicology, Department of Pharmacology, School of Medicine, University of California San Diego, La Jolla, California, USA; 2 Department of Medicine, School of Medicine, University of California San Diego, La Jolla, California, USA

**Keywords:** Uridine 5′-diphospho-glucuronosyltransferase (UDP-glucuronosyltransferase), oxidative stress, nuclear factor erythroid-derived 2-like 2 (NFE2L2) (Nrf2), intestinal metabolism, cytochrome P450 (CYP), animal model, pregnane X receptor (PXR), arsenic, bilirubin, intestinal epithelial cell (IEC) maturation, CAR, constitutive androstane receptor, FXR, farnesoid X receptor, Gsta1, glutathione S-transferase, *hUGT1*, humanized *UGT1* mice, iAs, inorganic arsenic, IEC, intestinal epithelial cell, NCoR1, nuclear receptor corepressor 1, Nqo1, NAD(P)H quinone dehydrogenase, NR, nuclear receptor, Nrf2, nuclear factor erythroid derived 2-like 2, PPARα, peroxisome proliferator-activator receptor alpha, PXR, pregnane X receptor, RNA-seq, RNA sequencing, ROS, reactive oxygen species, RT-qPCR, reverse transcription–quantitative polymerase chain reaction, TSB, total serum bilirubin, UGT1A1, UDP-glucuronosyltransferase 1

## Abstract

Inorganic arsenic (iAs) is an environmental toxicant that can lead to severe health consequences, which can be exacerbated if exposure occurs early in development. Here, we evaluated the impact of oral iAs treatment on UDP-glucuronosyltransferase 1A1 (UGT1A1) expression and bilirubin metabolism in humanized *UGT1* (*hUGT1*) mice. We found that oral administration of iAs to neonatal *hUGT1* mice that display severe neonatal hyperbilirubinemia leads to induction of intestinal UGT1A1 and a reduction in total serum bilirubin values. Oral iAs administration accelerates neonatal intestinal maturation, an event that is directly associated with UGT1A1 induction. As a reactive oxygen species producer, oral iAs treatment activated the Keap-Nrf2 pathway in the intestinal tract and liver. When Nrf2-deficient *hUGT1* mice (*hUGT1/Nrf2*^*−/−*^) were treated with iAs, it was shown that activated Nrf2 contributed significantly toward intestinal maturation and UGT1A1 induction. However, hepatic UGT1A1 was not induced upon iAs exposure. We previously demonstrated that the nuclear receptor PXR represses liver UGT1A1 in neonatal *hUGT1* mice. When PXR was deleted in *hUGT1* mice (*hUGT1/Pxr*^*−/−*^), derepression of UGT1A1 was evident in both liver and intestinal tissue in neonates. Furthermore, when neonatal *hUGT1/Pxr*^*−/−*^ mice were treated with iAs, UGT1A1 was superinduced in both tissues, confirming PXR release derepressed key regulatory elements on the gene that could be activated by iAs exposure. With iAs capable of generating reactive oxygen species in both liver and intestinal tissue, we conclude that PXR deficiency in neonatal *hUGT1/Pxr*^*−/−*^ mice allows greater access of activated transcriptional modifiers such as Nrf2 leading to superinduction of UGT1A1.

Inorganic arsenic (iAs) is a naturally occurring environmental element (1, 2). Based on the maximum permissible limit of 10 ppb recommended by the World Health Organization (3, 4), nearly 108 countries are affected by iAs contamination in groundwater. The most affected countries are in Southeast Asia, including Bangladesh, India, and Pakistan (5, 6). In the United States, approximately 13 million Americans are exposed to elevated levels of iAs. Even if iAs-safe drinking water is supplied, iAs in the soil and water used for irrigation can enter the food chain through crops and fodders (7). Many foods, including apple juice, chicken, wine, beer, and rice have been documented to have detectable levels of iAs. Rice accumulates more iAs than other food crops, with high levels of iAs detected in many rice-based products, such as rice milk, rice-based breakfast cereal, and infant rice cereal (8, 9, 10). Rice consumption has become the single biggest food source of human exposure to iAs (11). Chronic iAs exposure has been associated with several cancers, such as lung, bladder, skin, and liver (12), along with many noncancer diseases, including diabetes, atherosclerosis, and nonalcoholic fatty liver disease (13, 14, 15). These events indicate that oral absorption of iAs can influence key pathophysiological events at the site of absorption such as the intestinal tract in addition to additional tissues where the properties of iAs can induce oxidative stress.

Human UDP-glucuronosyltransferase 1A1 (UGT1A1) is the sole enzyme capable of conjugating and detoxifying bilirubin (16). Humanized *UGT1* mice (*hUGT1*) express the entire human *UGT1* locus under a mouse *Ugt1*-null background (*TgUGT1/Ugt1*^*−/−*^ mice) (17). The absence of murine *Ugt1a1* and the delayed expression of the human *UGT1A1* transgene results in severe neonatal hyperbilirubinemia in *hUGT1* mice during the suckling period (18, 19). In *hUGT1* mice, activation of the pregnane X receptor (PXR), constitutive androstane receptor (CAR), peroxisome proliferator-activator receptor alpha (PPARα), nuclear factor erythroid derived 2-like 2 (Nrf2), aryl hydrocarbon receptor, liver X receptor-α, and farnesoid X receptor (FXR) have been confirmed to regulate the *UGT1A1* gene in either the intestinal tract or liver in neonates leading to the metabolism of serum bilirubin (20, 21, 22, 23, 24, 25, 26).

The intestinal tract displays a unique physiology through repression that has been shown to play an important role in regulation of the *UGT1A1* gene. Gene repression is largely achieved by forming corepressor complexes, which are recruited to DNA by nuclear receptors and act through epigenetic modifications to limit nucleosomal DNA accessibility and transcriptional activation (27). Nuclear receptor corepressor 1 (NCoR1) was first discovered by its repressive function and highlighted by its important role in development (28, 29). When NCoR1 was selectively deleted in the intestinal tract of *hUGT1* mice (*hUGT1/Ncor1*^*ΔIEC*^), this event stimulated intestinal epithelial cell (IEC) maturation in newborns that led to derepression (*i.e.*, induction) of the *UGT1A1* gene and the resulting metabolism of serum bilirubin (20). In addition, obeticholic acid, an FXR agonist, when given orally to newborn *hUGT1* mice activates FXR target genes in both the intestinal tract and liver (26). The induction of UGT1A1 by obeticholic acid in intestines was dependent upon CAR as well as IEC maturation (26). Other prooxidants, such as cadmium and isothiocyanates (25, 30), activate CAR in the intestines of *hUGT1* neonates while inducing UGT1A1 (25), with isothiocyanates also inducing IEC maturation. While it has become clear that the processes leading to neonatal IEC maturation are linked to induction of intestinal UGT1A1, the generation of reactive oxygen species (ROS) and the activation of IEC maturation may be linked to a family of selective nuclear receptors.

One of the more potent producers of oxidative stress and ROS is iAs (31, 32). Of importance, as we will address, acute oral iAs exposure has a profound effect on the expression of proteins both in the intestinal tract and liver that have been linked to the metabolism of circulating bilirubin and induction of UGT1A1. Analysis of these events has uncovered an important linkage between iAs-induced IEC maturation and the role of Nrf2 and PXR in induction of intestinal and liver UGT1A1.

## Results

### Tissue-specific and developmental-dependent induction of human UGT1A1 by iAs in *hUGT1* neonatal mice

**Figure 1:**
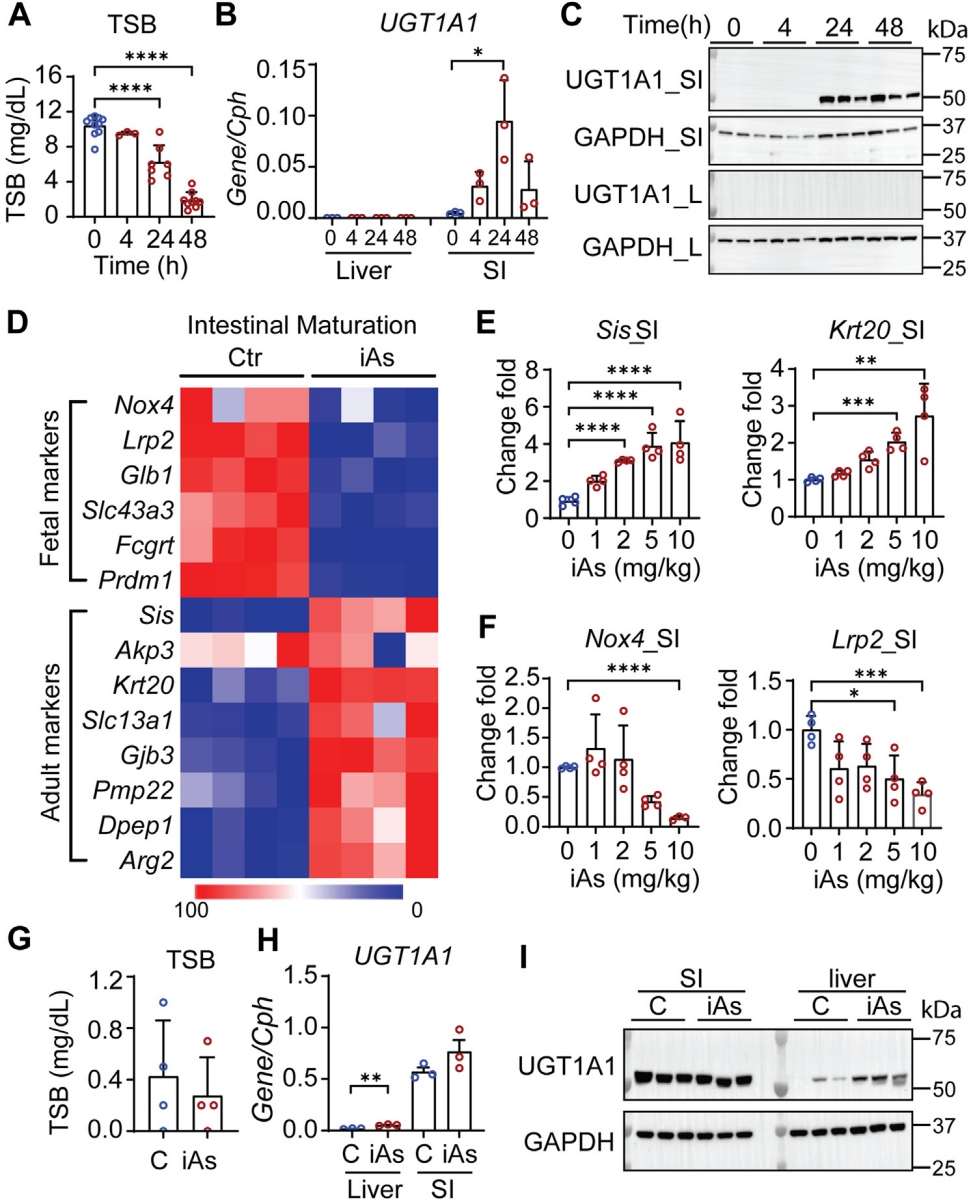
**Inorganic arsenic (iAs) exposure lowers total serum bilirubin (TSB) levels with intestinal UGT1A1 induction in *hUGT1* neonates.***A–F*, for time-dependent studies, neonatal *hUGT1* mice at 12 or 13 days old were orally treated with 10 mg/kg iAs. Mice were sacrificed at 4, 24, or 48 h post treatment. Liver, small intestine, and blood samples were collected. For dose-dependent studies, 13-day-old mice were orally treated with 1, 2, 5, or 10 mg/kg iAs. Tissue samples were collected at 24 h after treatment. *A*, TSB levels in time-dependent studies (n = 10, 3, 7, 9). *B*, RT-qPCR of *UGT1A1* in both liver and small intestine (SI) (n = 3). *C*, Western blot analysis of UGT1A1. GAPDH served as a loading control. *D*, RNA-Seq study. Small intestines were collected 24 h after iAs treatment. Samples from three mice were combined as one sample, with four samples from each group. Heatmap comparing genes related to small intestine maturation. *E*, RT-qPCR of small intestinal maturation markers, *Sis* and *Krt20* (n = 4). *F*, RT-qPCR of *Nox4* and *Lrp2* (n = 4). *G–I*, adult *hUGT1* mice at 8 weeks old were orally treated with 10 mg/kg iAs. Twenty-four hours later, tissue and blood were collected. *G*, TSB levels (n = 4). *H*, RT-qPCR of UGT1A1 in both liver and small intestine (n = 3). *I*, Western blot analysis. Results are described as mean ± SD (n ≥ 3). ∗*p* < 0.05, ∗∗*p* < 0.01, ∗∗∗*p* < 0.001, ∗∗∗∗*p* < 0.0001, Student’s *t* test. Individual *p* values were listed in Table S2. RT-qPCR, reverse transcription–quantitative polymerase chain reaction.

Humanized *UGT1* mice at ∼12 to 13 days old were administered a single oral dose of iAs at 10 mg/kg, which led to a time-dependent reduction in total serum bilirubin (TSB) levels at 4, 24, and 48 h post treatment (Fig. 1*A*). When we treated *hUGT1* neonates with a range of iAs concentrations from 1 to 10 mg/kg, a dose-dependent reduction of TSB levels was observed after 24 h (Fig. S1*A*). Our previous studies have linked serum TSB levels in *hUGT1* mice directly with the expression of hepatic and/or intestinal human UGT1A1 (17, 18). Oral iAs exposure led to the induction of UGT1A1 only in intestinal tissue (Figs. 1, *B* and *C* and S1*B*). Other human UGT1A isoforms quantitated by reverse transcription–quantitative polymerase chain reaction (RT-qPCR) confirmed that the *UGT1A3*, *-4/5*, *-6*, *-7*, and *-9/10* genes were also induced in the small intestine (Fig. S1*C*). It has been demonstrated that induction of intestinal UGT1A1 expression in neonatal *hUGT1* mice is positively correlated with IEC growth and enterocyte maturation (20, 26). When we performed RNA sequencing (RNA-seq) analysis on intestinal RNA from vehicle- and iAs-treated mice, genes expressed predominantly in fetal and newborn mice were all repressed to adult levels, while underexpressed adult IEC marker genes were all induced (Fig. 1*D*). Several of these maturation markers were confirmed by RT-qPCR analysis (Fig. 1, *E* and *F*). In addition, it has been demonstrated that selective induction of intestinal UGT1A1 by oral iAs is developmentally regulated. When adult *hUGT1* mice (8 weeks) were orally treated with iAs, baseline levels of intestinal UGT1A1 expression were not altered (Fig. 1, *G*–*I*). However, liver UGT1A1 expression was induced. The selective induction of liver UGT1A1 in adult mice and not neonates confirms that liver *UGT1A1* gene expression in newborn mice is repressed and this repression is developmentally regulated.

### iAs treatment alters nuclear receptor target genes

**Figure 2:**
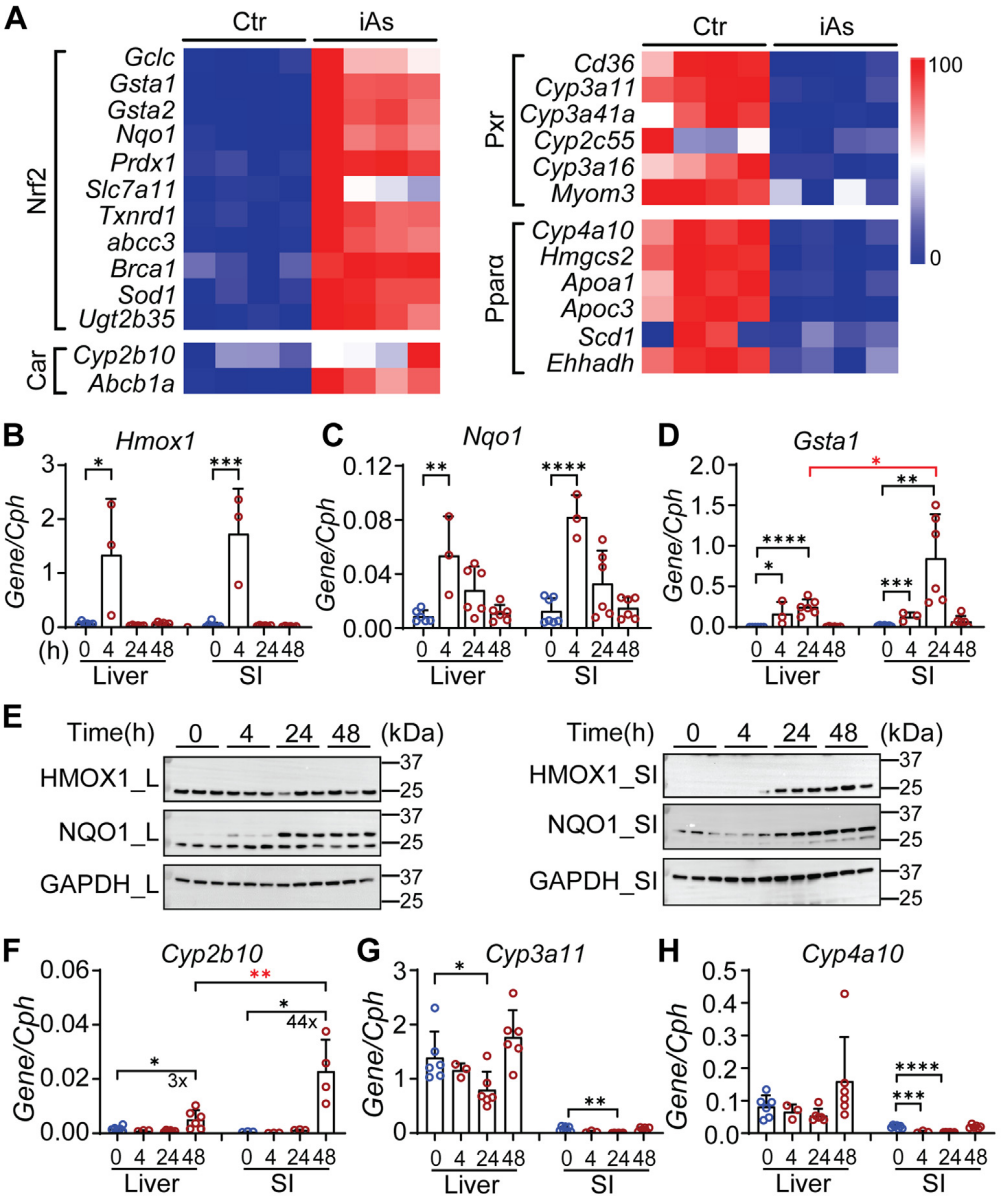
**iAs exposure induces oxidative stress and alters genes encoding drug metabolism enzymes.***A*, RNA-seq analysis. Neonatal *hUGT1* mice were treated with 10 mg/kg iAs and samples collected at 24 h. Heatmap analysis comparing genes that are regulated by different nuclear receptors. *B–D*, reverse transcription–quantitative polymerase chain reaction of oxidative stress markers including *Hmox1*, *Nqo-1*, and *Gsta1* (n = 6, 3, 6, 6). *E*, Western blot analysis of HMOX1 and NQO1 in both liver (L) and small intestine (SI). *F–H*, RT-qPCR of *Cyp2b10*, *Cyp3a11*, and *Cyp4a10* in both liver and small intestine (n = 6, 3, 6, 6). Results are described as mean ± SD. ∗*p* < 0.05, ∗∗*p* < 0.01, ∗∗∗*p* < 0.001, ∗∗∗∗*p* < 0.0001, Student’s *t* test. Individual *p* values were listed in Table S3.

An important property of iAs and one of the most intensely studied mechanisms leading to carcinogenesis and other health effects is the production of ROS. The production of ROS can lead to the activation of Nrf2, a master regulator that responds to oxidative stress. The production of oxidative stress and activation of the Keap1-Nrf2 pathway promotes the overexpression of antioxidant enzymes following binding of Nrf2 to antioxidant response elements, resulting in transcriptional activation (31, 33). Following iAs treatment, a large panel of genes regulated by Nrf2 were significantly induced (Fig. 2*A*). Further confirmation for induction of antioxidant responsive genes known to be activated following ROS production, heme oxygenase I (*Hmox1*), NAD(P)H quinone dehydrogenase 1 (*Nqo1*), and glutathione S-transferase alpha 1 (*Gsta1*) were induced in both liver and small intestines (Fig. 2, *B*–*E*). The induction of these three genes followed a remarkably similar pattern in liver and intestinal tissues, although the induction of *Gsta1* was higher in small intestine (*p* < 0.05) (Fig. 2*D*). HMOX1 and NQO1 were induced in both liver and small intestines (Fig. 2*E*). In addition to the expression of Nrf2 target genes, expression of the *Cyp2b10* gene, a target of activated CAR, was induced in both liver and intestinal tissues, with a much higher induction at approximately 44-fold in small intestine in comparison with a 6-fold increase in liver (Fig. 2*F*). The induction of *Cyp2b10* is indicative of oxidative stress, since it has previously been confirmed that agents such as isothiocyanates and cadmium, which produce oxidative stress, can activate CAR in a non-Nrf2-dependent fashion (25). Further analysis of RNA-seq studies revealed that nuclear receptors PXR and PPARα target genes that are uniquely repressed following iAs treatment (Fig. 2*A*). This can also be observed with RT-qPCR analysis of intestinal *Cyp3a11* expression, which is regulated by PXR, and *Cyp4a10*, which is regulated by PPARα (Fig. 2, *G* and *H*). Clearly, iAs can differentially regulate downstream nuclear receptor (NR) target genes either by activation of the receptors by oxidative stress or by inhibition.

### The role of Nrf2 in iAs-induced *UGT1A1* gene expression

**Figure 3:**
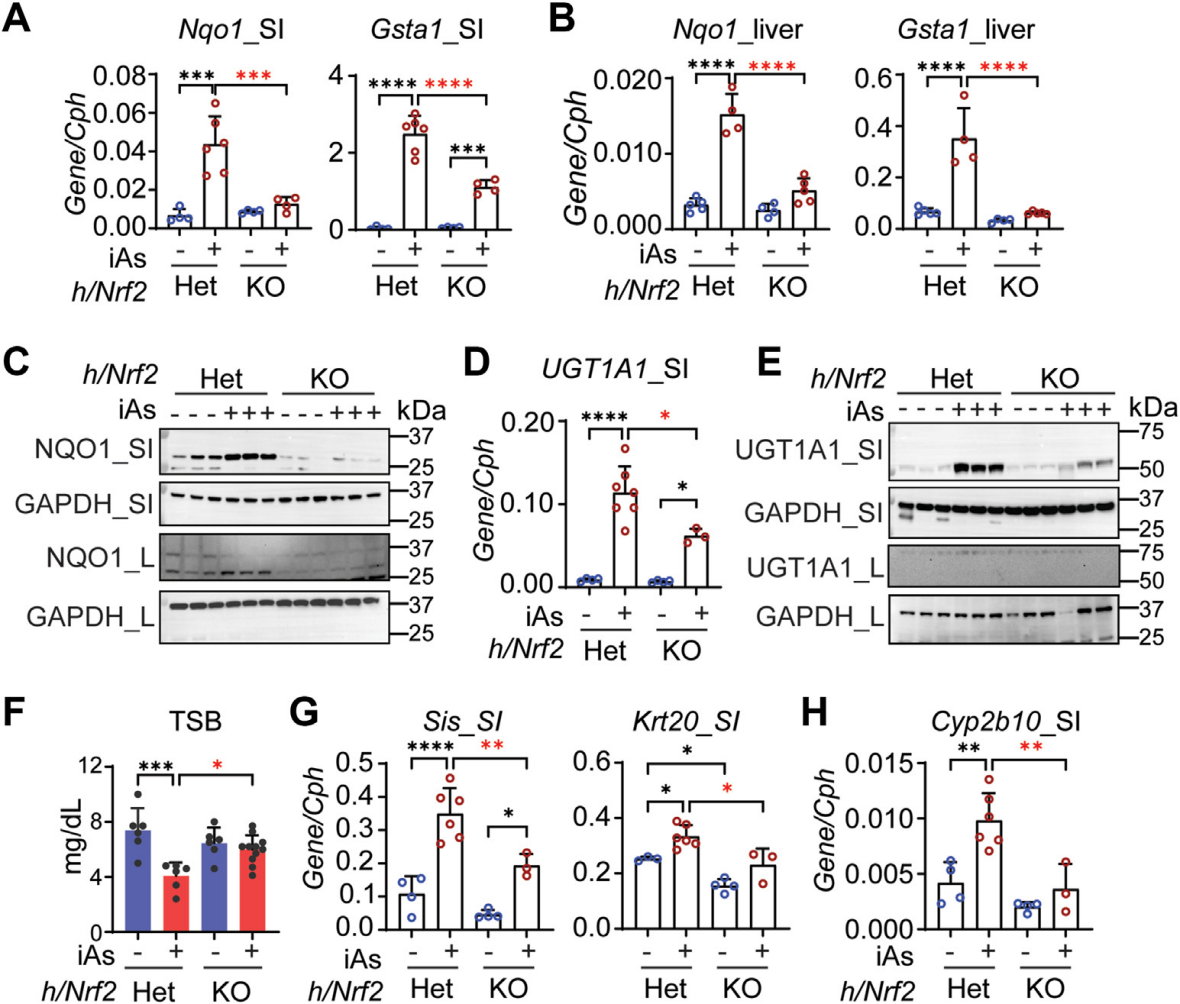
**The dependency of Nrf2 toward iAs-mediated induction of UGT1A1.***hUGT1/Nrf2*^*+/−*^ and *hUGT1/Nrf2*^*−/−*^ neonates at 13 days old were orally treated with vehicle or 10 mg/kg iAs. Small intestines and blood samples were collected 24 h post treatment. *A*, RT-qPCR of Nrf2 target genes including *Nqo-1* and *Gsta1* in small intestines (SI, n = 4, 6, 4, 4) and liver (L, n = 5, 4, 4, 5). *B* and *C*, following iAs treatment, Western blot analysis of NQO1 in SI and liver L. *D*, RT-qPCR of *UGT1A1* gene expression in SI (n = 4, 6, 4, 3). *E*, Western blot analysis of UGT1A1 in SI and L. *F*, total serum bilirubin (TSB) levels (n = 6, 6, 6, 11). *G*, RT-qPCR of *Sis* and *Krt20* gene expression in SI (n = 4, 6, 4, 3). *H*, RT-qPCR analysis of *Cyp2b10* gene expression in SI (n = 4, 6, 4, 3). Results are described as mean ± SD. ∗*p* < 0.05, ∗∗*p* < 0.01, ∗∗∗*p* < 0.001, ∗∗∗∗*p* < 0.0001, one-way ANOVA. Individual *p* value were listed in Table S4. RT-qPCR, reverse transcription–quantitative polymerase chain reaction.

Newborn *hUGT1/Nrf2*^*+/−*^ and *hUGT1/Nrf2*^*−/−*^ mice develop severe neonatal hyperbilirubinemia like that observed in newborn *hUGT1* mice. *hUGT1/Nrf2*^*−/−*^ and *hUGT1/Nrf2*^*+/−*^ mice at 13 days old were treated with iAs by oral gavage. In both liver and intestinal tissue, gene expression of the Nrf2 target genes, *Nqo1* and *Gsta1*, were either abolished or significantly reduced in small intestine and liver of *hUGT1/Nrf2*^*−/−*^ mice (Fig. 3, *A*–*C*). In the small intestine, *UGT1A1* gene and protein expression was induced following iAs treatment in *hUGT1/Nrf2*^*+/−*^ mice but was greatly reduced in iAs-treated *hUGT1/Nrf2*^*−/−*^ mice (Fig. 3, *D* and *E*). The other genes associated with the *UGT1* locus were also repressed following iAs treatment in *hUGT1/Nrf2*^*−/−*^ mice (Fig. S2*A*). The pattern of intestinal UGT1A1 expression was reflected in TSB values, with a reduction in *hUGT1/Nrf2*^*+/−*^ mice followed by little change in *hUGT1/Nrf2*^*−/−*^ mice (Fig. 3*F*). It should also be noted that Nrf2 deficiency led to reduced iAs-stimulated intestinal maturation as measured by a reduction in intestinal maturation gene expressions (Fig. 3*G*). The reduction in intestinal maturation can be reflected in reduced intestinal UGT1A1 expression.

There was little impact of Nrf2 deficiency on the inhibition of *Cyp4a10* and *Cyp3a11* in the small intestines (Fig. S2*B*), leading us to conclude that iAs and not ROS production inhibits directly PXR and PPARα. However, iAs treatment and ROS production are linked to induction of the *Cyp2b10* gene through an Nrf2-dependent process (Fig. 3*H*). iAs-initiated induction of *Cyp2b10* gene expression is greatly reduced in *hUGT1/Nrf2*^*−/−*^ mice.

**Figure 4:**
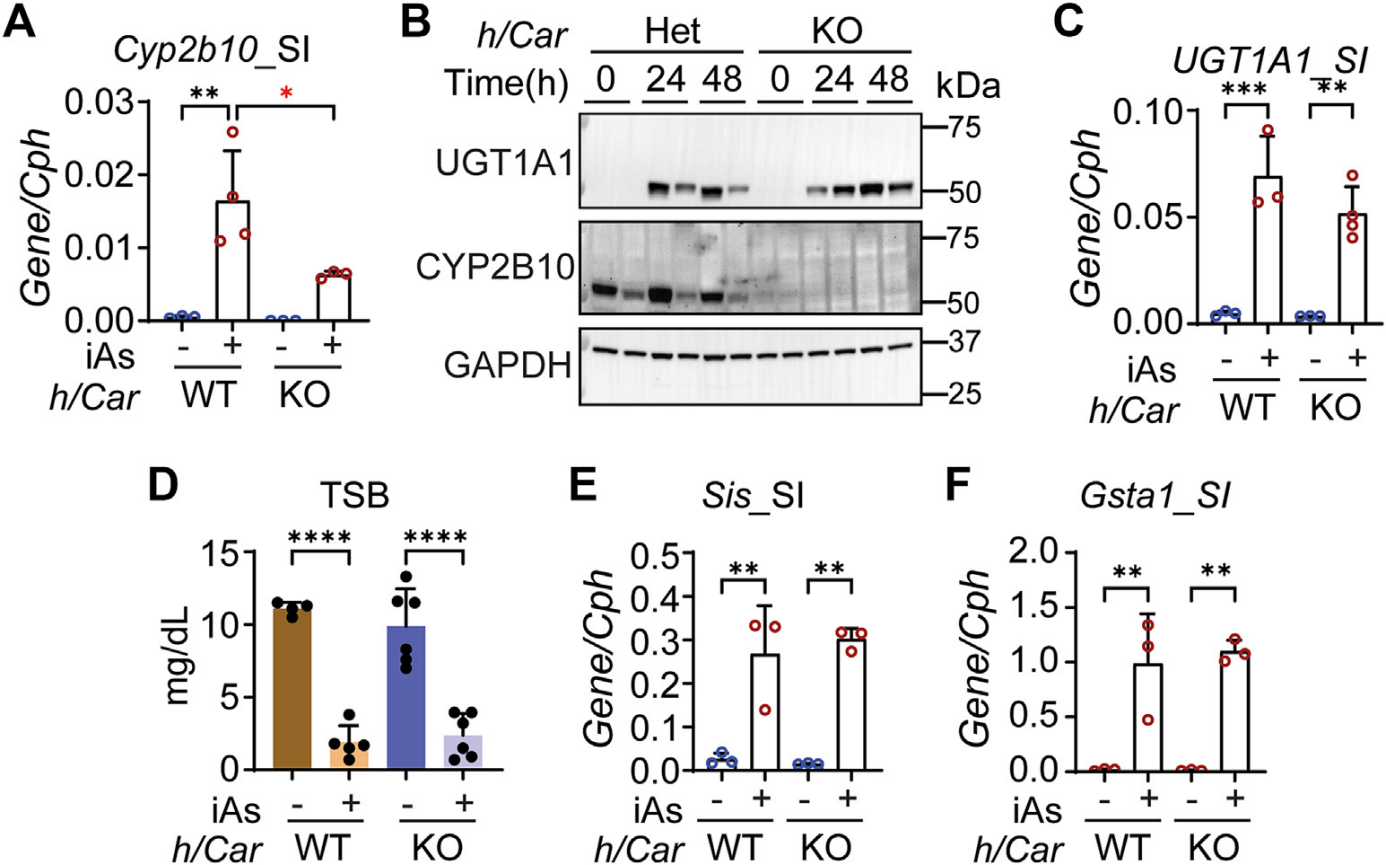
**iAs-mediated UGT1A1 induction is independent from CAR.***hUGT1/Car*^*+/−*^ and *hUGT1/Car*^*−/−*^ neonatal mice at 12 days old were orally treated with 10 mg/kg iAs for 48 h. *A*, RT-qPCR of *Cyp2b10* gene expression (n = 3, 4, 3, 3). *B*, Western blot analysis of UGT1A1 and CYP2B10 in the small intestine (SI). *C*, RT-qPCR analysis of *UGT1A1* gene expression in SI (n = 3, 3, 3, 4). *D*, total serum bilirubin (TSB) levels (n = 4, 5, 6, 6). *E* and *F*, RT-qPCR of *Sis* and *Gsta1* gene expression (n = 3). Results are described as mean ± SD. ∗*p* < 0.05, ∗∗*p* < 0.01, ∗∗∗*p* < 0.001, ∗∗∗∗*p* < 0.0001, one-way ANOVA. Individual *p* values were listed in Table S5. RT-qPCR, reverse transcription–quantitative polymerase chain reaction.

To determine if this process is associated with CAR, we treated neonatal *hUGT1/Car*^*−/−*^ and WT mice with iAs. Expression of the *Cyp2b10* gene in small intestines was blocked in *hUGT1/Car*^*−/−*^ mice, with undetectable levels of induced protein (Fig. 4, *A* and *B*). This result establishes an important link between iAs exposure, Nrf2, and CAR. Although UGT1A1 can be induced by activated CAR (30), deletion of CAR had no impact on *UGT1A1* gene or protein expression in the small intestines following iAs exposure (Fig. 4, *B*–*D*). We can speculate that activation of Nrf2 by iAs production of ROS is sufficient for maximal transcriptional activation of the *UGT1A1* gene. Alternatively, induction of UGT1A1 in *hUGT1/Car*^*−/−*^ mice may result from intestinal maturation and ROS as indicated by induction of the enterocyte *Sis* (Fig. 4*E*) and *Gsta1* genes (Fig. 4*F*), both in WT and CAR-deficient mice.

### Contribution of PXR and PPARα toward iAs-induced expression of UGT1A1

RNA-seq analysis of intestinal RNA following iAs exposure revealed that target genes regulated by PXR and PPARα are inhibited (Fig. 2*A*). Activation of both PXR and PPARα in *hUGT1* mice leads to induction of UGT1A1 in liver and intestines of adult mice (18, 23). To examine the contribution of PXR and PPARα toward expression of UGT1A1 following iAs exposure, we deleted PXR (*hUGT1/Pxr*^*−/−*^) and PPARα (*hUGT1/Pparα*^*−/−*^) followed by exposure of 13-day-old neonates to oral iAs. iAs exposure of *hUGT1/Pparα*^*+/−*^ and *hUGT1/Pparα*^*−/−*^ neonates resulted in UGT1A1 induction in both strains (Fig. S3, *A–C*), demonstrating that PPARα expression is not tied to UGT1A1 induction.

**Figure 5:**
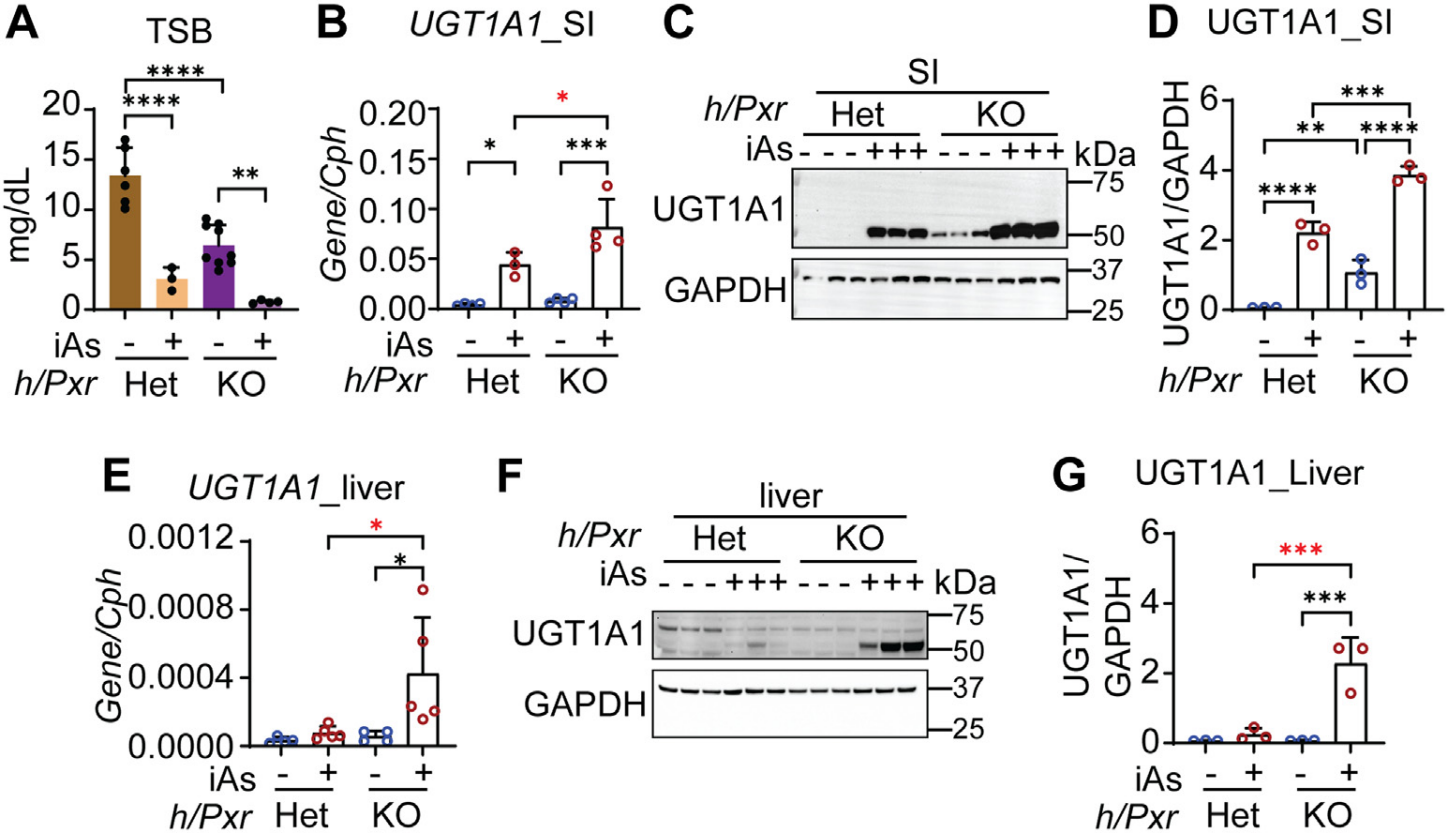
**PXR repression contributes to iAs-mediated induction of UGT1A1.***hUGT1/PXR*^*+/−*^ and *hUGT1/PXR*^*−/−*^ 12-day-old neonates were orally treated with 10 mg/kg iAs. Small intestines, liver, and blood samples were collected at 48 h after treatment. *A*, total serum bilirubin (TSB) levels (n = 6, 3, 9, 4). *B*, RT-qPCR analysis of intestinal *UGT1A1* gene expression (n = 4, 3, 4, 4). *C*, Western blot analysis of intestinal UGT1A1. *D*, quantification of the Western blot in *C*. *E*, RT-qPCR analysis of liver *UGT1A1* gene expression (n = 4, 5, 4, 5). *F*, Western blot analysis of liver UGT1A1 *G*, quantification of Western blot in *F*. Results are described as mean ± SD. ∗*p* < 0.05, ∗∗*p* < 0.01, ∗∗∗*p* < 0.001, ∗∗∗∗*p* < 0.0001, one-way ANOVA. Individual *p* values were listed in Table S6. RT-qPCR, reverse transcription–quantitative polymerase chain reaction.

However, PXR plays an important repressive role in both the intestinal tract and liver. In *hUGT1/Pxr*^*−/−*^ mice, TSB levels in newborn mice are 50% lower than in *hUGT1/Pxr*^*+/−*^ neonates (Fig. 5*A*), resulting from elevated constitutive expression confirmed by RT-qPCR levels (Fig. 5*B*) and Western blot analysis of intestinal UGT1A1 (Fig. 5, *C* and *D*). In *hUGT1/Pxr*^*+/−*^ neonates, there is no induction of UGT1A1 in liver tissue (Fig. 5, *E*–*G*). However, in *hUGT1/Pxr*^*−/−*^ mice, the actions of iAs lead to induction of liver UGT1A1 (Fig. 5, *E*–*G*). Analysis of small intestine UGT1A1 expression following iAs treatment in *hUGT1/Pxr*^*−/−*^ mice shows superinduction compared with induction in *hUGT1/Pxr*^*+/−*^ neonates (Fig. 5, *C* and *D*). Other human UGT1A isoforms quantitated by RT-qPCR confirmed that *UGT1A3*, *-4/5*, *-6*, and *-9/10* were also induced in the small intestine in *hUGT1/Pxr*^*−/−*^ mice (Fig. S4). These findings confirm that PXR expression in both liver and intestinal tract in neonatal mice serves to repress *UGT1A1* gene expression. The absence of PXR leads to derepression, and in the presence of iAs, the epigenetic changes associated with the *UGT1A1* gene in both liver and intestines provides access to activated transcriptional events induced by oxidative stress.

## Discussion

In newborn children and *hUGT1* neonatal mice, the *UGT1A1* gene is repressed in the liver leading to increases in TSB levels, which results in varying degrees of hyperbilirubinemia (19, 34). In humans, there are few reports of the developmental properties of UGT1A1 in neonatal liver and virtually no information on the importance of intestinal UGT1A1 expression as a modulator of neonatal hyperbilirubinemia. The expression of UGT1A1 and the other *UGT1A* genes is regulated in a tissue-specific fashion similar to expression patterns confirmed in human tissues (35, 36, 37, 38, 39, 40). Of importance, the role of the intestinal tract and activation of the *UGT1A1* gene in controlling the onset and development of neonatal hyperbilirubinemia has been clearly documented (26, 41). While there is significant information on the mechanistic events leading to induction and developmental expression of UGT1A1 in *hUGT1* mice following activation of the family of NRs (22, 23, 24, 42), we have linked the production of intracellular ROS by iAs exposure to neonatal mice as a catalyst for intestinal *UGT1A1* gene expression. Although iAs-induced ROS is produced both in the intestinal tract and liver of neonates, induction of UGT1A1 could only be detected in the intestinal tract. This finding indicates that unique physiological events associated with the developing intestinal tract underlie the mechanisms associated with iAs induction of intestinal UGT1A1.

Along with induction of intestinal UGT1A1, iAs treatment drove the induction of adult intestinal maturation markers from their resting fetal levels to adult levels, while inhibiting the induction of fetal maturation markers. The increase in IEC maturation following iAs treatment and the induction of UGT1A1 resembles similar results when intestinal NCoR1 is selectively deleted from the intestinal tract in *hUGT1* neonates. During development of the intestinal tract in neonatal mice, NCoR1 serves to suppress IEC maturation (20). The repressive nature of NCoR1 and the NRs is controlled through specific phosphorylation events (43, 44, 45, 46), with a host of different kinases implicated in NCoR1 regulation. Phosphorylation of NCoR1 uncouples it from the NRs, resulting in derepression of transcription. Oral iAs treatment of neonatal *hUGT1* mice activates stress-linked proteins such as p38-mitogen-activated protein kinase and extracellular signal-regulated kinase (ERK) 1/2 (47, 48, 49). If NCoR1 is targeted in the intestinal tract by activated p38 or ERK1/2, its phosphorylation would result in derepression of IEC maturation as well as the *UGT1A1* gene. While the functional role of NCoR1 may be important in iAs-induced IEC maturation and expression of UGT1A1, part of the actions of the intestinal tract following exposure can be linked to regulation of NRs, several of which are associated with repression of UGT1A1.

The actions of oral iAs exposure of *hUGT1* neonatal mice clearly resulted in induction of ROS-dependent Nrf2 target genes in addition to that of CAR-dependent target genes. In Nrf2-deficient mice, induction of intestinal and liver *Nqo1*/Nqo1 expression by iAs was completely suppressed, verifying that iAs-induced ROS drives oxidative stress. In the small intestines, there is dramatic but not total reduction in iAs-induced UGT1A1, implicating that additional Nrf2-independent processes are in play to regulate *UGT1A1* gene expression. It is also clear that deletion of Nrf2 reduces iAs induction of the intestinal maturation markers, a finding that ties oxidative stress and Nrf2 activation to control of IEC maturation in neonates. Our findings also demonstrate that iAs exposure induces intestinal *Cyp2b10*/CYP2B10, which is highly dependent upon Nrf2. Interestingly, when we deleted CAR in *hUGT1* mice (*hUGT1/Car*^*−/−*^), iAs induction of CYP2B10 was virtually eliminated since CAR plays a major role in the regulation of *Cyp2b10*. However, CAR deficiency had no impact on the induction of *UGT1A1*/UGT1A1. While activated CAR and Nrf2 can induce both *UGT1A1* and *Cyp2b10* genes in *hUGT1* mice, the lack of iAs-initiated induction of CYP2B10 in *hUGT1/Car*^*−/−*^ mice demonstrates that the generation of ROS catalyzes an important association between CAR and Nrf2 that is required to induce the *Cyp2b10* gene.

From RNA-seq analysis and reverse genetic studies, induction of oxidative stress by iAs exposure links activation of Nrf2 and IEC maturation to events leading to induction of intestinal UGT1A1. Also apparent in these studies was an inhibitory pattern that impacted PXR and PPARα target genes. This finding was relevant since we had previously demonstrated that deletion of PXR in *hUGT1* mice led to derepression of hepatic UGT1A1 in neonates (23), while NCoR1-deficient *hUGT1* neonates resulted in an activated gene expression profile linked to PPARα (20). Thus, inhibition of NRs such as PXR and PPARα by iAs exposure may directly impact expression of UGT1A1. To examine this possibility, PXR and PPARα were deleted in *hUGT1*, and the *hUGT1/Pxr*^*−/−*^ and *hUGT1/Pparα*^*−/−*^ neonates exposed to iAs. While there was little impact on iAs-induced expression of UGT1A1 in *hUGT1/Pparα*^*−/−*^ mice, there was a dramatic impact in *hUGT1/Pxr*^*−/−*^ mice that influenced expression of UGT1A1 in both the intestinal tract and liver. Deletion of PXR led to significant derepression of UGT1A1 in the small intestine. While iAs treatment of *hUGT1/Pxr*^*+/−*^ led to induction of UGT1A1 in the intestines, exposure of PXR knockout mice led to far greater UGT1A1 induction or superinduction. The treatment of wildtype or *hUGT1/Pxr*^*+/−*^ mice with iAs did not result in UGT1A1 induction in liver, although it did result in hepatic oxidative stress and induction of Nrf2 target genes. When we examined liver tissue from iAs-treated *hUGT1/Pxr*^*−/−*^ mice, there was significant induction of UGT1A1. It is worth noting that the PXR binding site (PXRE) and the Nrf2 binding antioxidant response elements are located within a 280-bp DNA region on the *UGT1A1* promoter (18, 42). The close proximity of these binding sites coupled with findings that iAs can induce hepatic UGT1A1 in *hUGT1/Pxr*^*−/−*^ mice allows us to conclude that PXR represses the *UGT1A1* gene by masking Nrf2-binding sites, and removal of PXR heightens the sensitivity toward ROS-activated Nrf2.

Oral exposure of neonatal *hUGT1* mice to iAs has uncovered unique signaling events that control developmental expression of the *UGT1A1* gene in intestines and liver but by uniquely different mechanisms. We have confirmed that the single commonality of iAs treatment between liver and intestines is the production of ROS and the activation of Nrf2. Although the mechanism is unclear, iAs exposure and the activation of Nrf2 in the intestines helps direct IEC maturation, which is directly linked to induction of UGT1A1. The activation of Nrf2 by iAs exposure in liver has minimal impact on UGT1A1 expression. However, in liver and intestines, PXR participates actively in repressing the developmental expression of UGT1A1. Since UGT1A1 is abundantly expressed in adult liver and intestinal tissue and is solely responsible for the metabolism and clearance of serum bilirubin, the developmental delay in liver and intestinal tissue can be directly linked to PXR, although the mechanism leading to the repressive actions toward the *UGT1A1* gene during development are unknown.

## Experimental procedures

### Animals and treatment

Transgenic mice expressing the human *UGT1* locus in a *Ugt1*^−/−^ background (*hUGT1*) were developed previously (17). The *Car*-null (*Car*^*−/−*^) mice were a gift from Dr Masahiko Negishi (National Institute of Environmental Health Sciences) and were crossed with *hUGT1* mice to generate *hUGT1/Car*^*−/−*^ mice. The *Pparα*^−/−^, *Pxr*^−/−^, and *Nrf2*^−/−^ mice were purchased from The Jackson Laboratory and were used to generate *hUGT1/Pparα*^*−/−*^, *hUGT1/Nrf2*^*−/−*^, and *hUGT1/Pxr*^*−/−*^ mice. All mouse strains were housed in a pathogen-free UCSD Animal Care Facility and received food and water *ad libitum*. All animal protocols were reviewed and approved by the UCSD Animal Care and Use Committee. In neonatal studies, male and female pups at ∼12 to 13 days old with body weight between 6.0 g and 9.0 g were used. Each experiment result was obtained from at least two different litters. In each litter, mice were randomly divided into control and treated groups. Littermate controls were used for all experiments. The sample size calculation was based on serum TSB levels from vehicle and treated mice at the beginning stage of the experiment. Experimental design assistant (https://eda.nc3rs.org.uk/eda/login/auth) was used as the power calculator. The power of the experiment was set to 90%, and the calculated N value for each group is 3. Therefore, we used at least three mice in each group in the following experiments.

Time- and dose-dependent studies were performed. For time-dependent studies, 12-day-old *hUGT1* neonatal mice were treated by oral gavage with water (vehicle) or 10 mg/kg sodium arsenite (iAs, Sigma-Aldrich, S7400) dissolved in vehicle. Blood and tissues were collected at 4, 24, or 48 h after administration. For dose-dependent studies, 13-day-old *hUGT1* mice were treated by oral gavage with water or 1, 2, 5, 10 mg/kg iAs. Blood and tissues were collected at 24 h after treatment. For the above transgenic neonatal mice, 12- or 13-day-old neonatal mice were treated by oral gavage with water or 10 mg/kg iAs. Blood and tissues were collected at 24 or 48 h after treatment. In adult studies, vehicle or 10 mg/kg iAs was orally administrated to 8-week-old male mice and tissues were collected at 24 h after iAs treatment. For tissue collection, liver and small intestine tissues were washed with ice-cold phosphate-buffered saline and then snap frozen in liquid nitrogen immediately. Tissue samples were stored at −80 °C until analysis.

### Reverse transcription–quantitative-PCR and RNA sequencing

Total RNA was extracted from the collected liver or small intestinal tissue samples using TRIzol reagent (Thermo Fisher Scientific, 15596026). The cDNA sample was generated using the iScript complementary DNA synthesis kit (Bio-Rad, 1708891) according to the manufacturer’s instructions. RT-qPCR was performed on a CFX96 qPCR system (Bio-Rad, C1000 Touch Thermal Cycler, CFX96 Real-Time System) using Ssoadvanced SYBR Green reagent (Bio-Rad, 1725274). All primers used in this study were purchased from Integrated DNA technology and reported in Table S1.

For RNA-seq, 13-day-old *hUGT1* mice were first treated with vehicle or iAs (10 mg/kg) for 24 h, and then small intestines were collected for total RNA isolation. The extracted RNA samples from three mice were combined to represent a single sample. For both vehicle and iAs treatment, RNA-Seq analysis was run on four samples (12 mice for each treatment). The sequencing library for each sample was prepared using the Illumina Stranded mRNA Saple Prep Kit (20040534; Illumina) with 1 μg of RNA. Using an Illumina NovaSeq6000 sequencer in the IGM Genomics Center at UCSD, samples were sequenced with Paired End 100 base pair sequencing, with base calling performed using bcl2fastq (v2.20; Illumina). STAR (v2.7.9a) was used to align the sequencing reads to the mouse genome (mm10) and to generate gene-level counts from uniquely aligned reads. Differentially expressed genes were calculated with DESeq2 (1.34.0).

### Bilirubin measurement

Blood samples were collected from the submandibular vein and centrifuged at 14,000*g* for 2 min to obtain serum. The total serum bilirubin (TSB) levels were measured using a Unistat Bilirubinometer (Reichert, Inc).

### Protein preparation and Western blot analysis

Tissue samples (100 mg) were homogenized in 0.4 ml 1 × RIPA lysis buffer (EMD Millipore, 20-188) containing a protease and phosphatase inhibitor cocktail (Thermo Fisher Scientific, 87786 and 78420) and incubated on ice for 30 min. The resultant mixture was centrifuged at 16,000*g* for 20 min at 4 °C, and the supernatants were transferred to a new tube and stored at −80 °C until analyzed.

Electrophoresis was performed by using NuPAGE 4 to 12% BisTris-polyacrylamide gels (Thermo Fisher Scientific, NW04127BOX) following the manufacturer’s instructions. Protein (30 μg) was subject to electrophoresis at 170 V for 1 h and transferred at 20 V for 1 h to PVDF membranes (EMD Millipore, IPVH00010). After washing, membranes were blocked with 5% nonfat milk at room temperature for 1 h and incubated with primary antibodies at 4 °C overnight. The following antibodies were used: rabbit antihuman UGT1A1 (Abcam, Ab-170858), mouse anti-GAPDH (Santacruz, sc-47724), rabbit anti-NQO1 (Cell Signaling Technology, CS62262), goat anti-SIS (Santacruz, sc-27603), rabbit anti-CYP2B10 (a kind gift from Dr Masahiko Negishi, NIEHS), and rabbit anti-HO1 (Cell Signaling Technology, 5853). After incubation with primary antibody, membranes were exposed to HRP-conjugated secondary antibodies (anti-mouse IgG, anti-rabbit IgG and anti-goat IgG, Cell Signaling Technology). All primary antibodies were diluted 1:1000 and secondary antibodies were diluted 1:3000. Protein was detected by the ECL Plus Western blotting detection system (Bio-Rad) and was visualized by the Bio-Rad Chemidoc Touch Imaging System. Band density was quantified by using Image Lab 5.2.1 software.

### Statistics

All results were subjected to statistical analysis. Student t test analyses (nonparametric Mann–Whitney test) were performed for two-group comparison. For comparison among multiple groups, one-way ANOVA with Tukey’s multiple comparison test was used. All statistics and graphs were generated using GraphPad Prism software (GraphPad Software). Data are expressed as means ± SD, and *p* values smaller than 0.05 were considered statistically significant. The results of all statistical comparisons between experimental groups are clearly indicated in each figure legend, with exact *p* values showed in Tables S2–S10.

## Data availability

All data are contained within the article.

## Supporting information

This article contains supporting information.

## Conflict of interest

The authors declare that they have no conflicts of interest with the contents of this article.
