# Supplementary material for: Oral arsenic administration to humanizedUDP-glucuronosyltransferase1 neonatal mice induces UGT1A1 through a dependence on Nrf2 and PXR
Source: J Biol Chem. 2023 Jan 30;299(3):102955. doi: 10.1016/j.jbc.2023.102955 (PMC9996368; doi:10.1016/j.jbc.2023.102955)
Supplement: Supplemental Figures S1–S4 and Tables S1–S10 [file mmc1.docx]

**SUPPORTING INFORMATION**

**Oral arsenic administration to *humanized UDP-glucuronosyltransferase 1* neonatal mice induces UGT1A1 through a dependence on Nrf2 and PXR**

Xiaojing Yang^1^, André A. Weber^1^, Elvira Mennillo^1^, Miles Paszek^1^, Samantha Wong^1^, Sabrina Le^1^, Jia Ying Ashley Teo^1^, Max Chang^2^, Christopher W. Benner^2^, Robert H. Tukey^1^, Shujuan Chen^1^

1 Laboratory of Environmental Toxicology, Department of Pharmacology, School of Medicine, University of California San Diego, La Jolla, CA 92093

2 Department of Medicine, School of Medicine, University of California San Diego, La Jolla, CA 92093

**Correspondence**: [s18chen@health.ucsd.edu](mailto:s18chen@health.ucsd.edu)

UC San Diego, 9500 Gilman Drive, La Jolla, CA 92093-0722

**Running Title**: Nrf2 and PXR regulate UGT1A1 in iAs exposed neonates


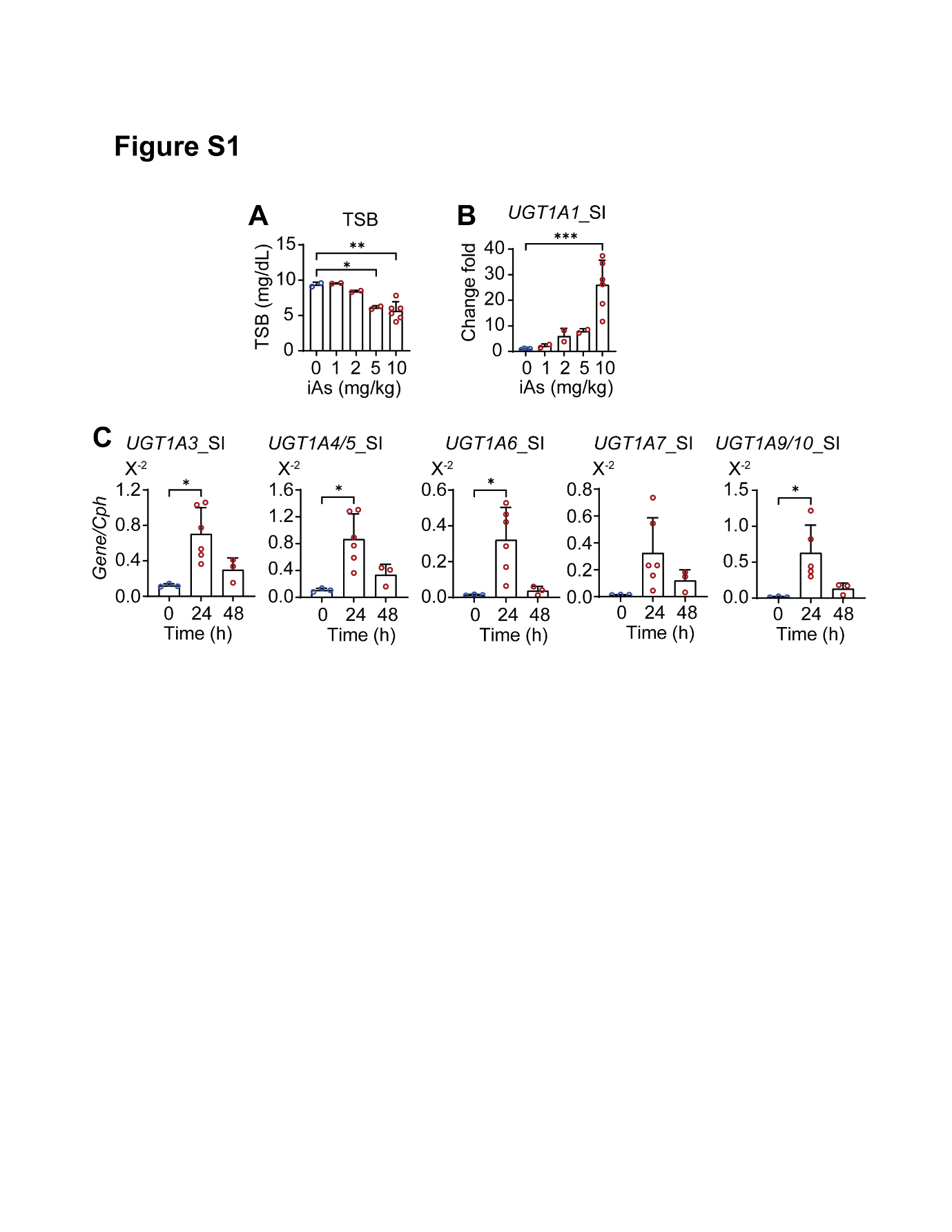


**Supporting Figure 1.** **Impact of iAs treatment on expression of the *UGT1* locus**. (**A**) 12-day-old *hUGT1* neonates were treated with different concentrations of iAs followed by analysis of TSB levels after 24 h (n=2, 2, 2, 2, 6). (**B**) Fold change in intestinal *UGT1A1* expression (n=4, 2, 2, 2, 6). (**C**) RT-qPCR analysis of the different *UGT1A* genes (n=3, 6, 3). Results are described as mean ± SD. *p<0.05, **p<0.01, ***p<0.001, ****p<0.0001, Student’s t test. Individual P value were listed in supporting information table S7.

**
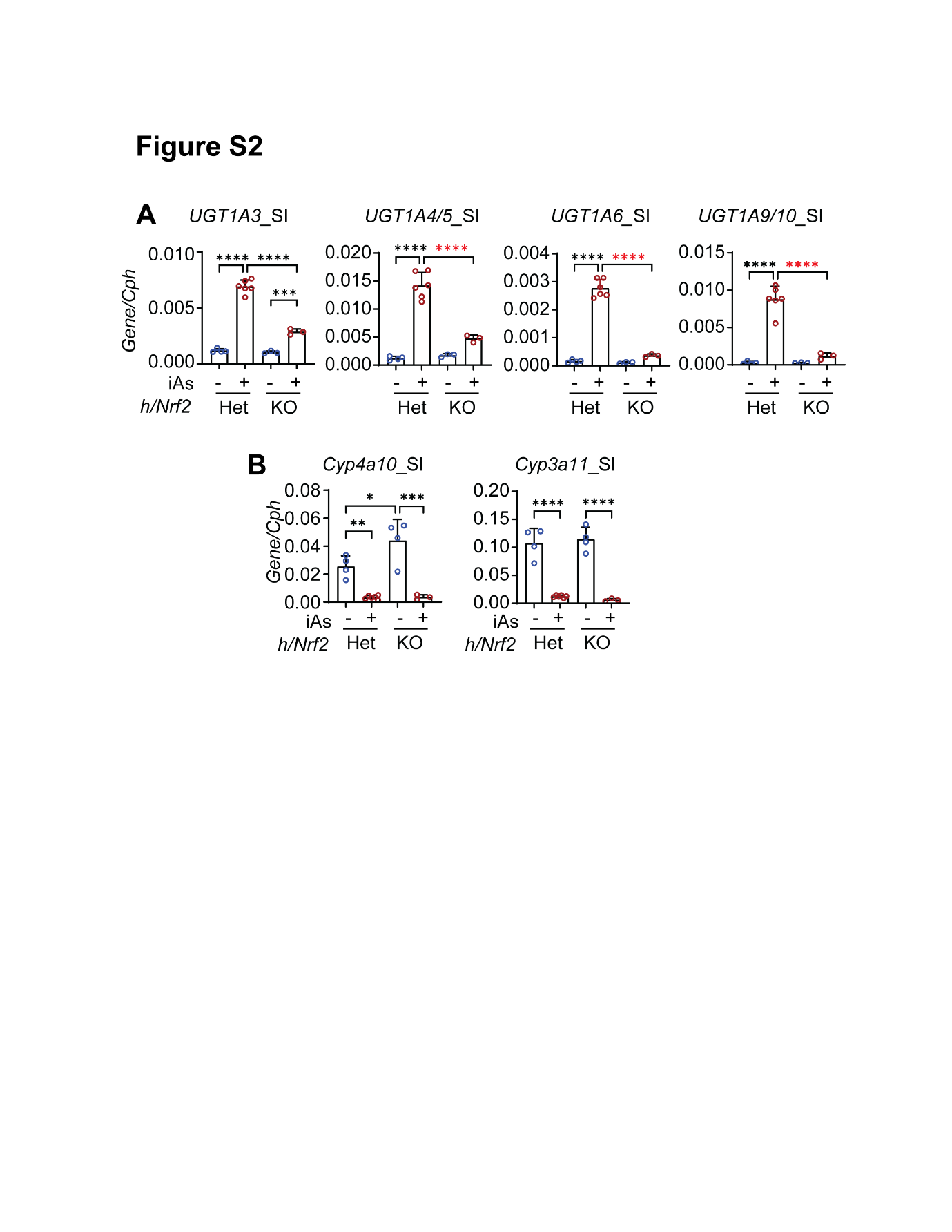
**

**Supporting Figure 2.** **The impact of Nrf2 on gene expression**. Control and *hUGT1/Nrf2^-/-^* 12-day-old neonates were orally treated with 10 mg/kg iAs and small intestines and liver were collected after 24h. (**A**) RT-qPCR analysis of the different *UGT1A* genes (n=4, 6, 3, 3). (**B**) RT-qPCR of *Cyp4a10* and *Cyp3a11* gene expression in small intestine (n=4, 6, 4, 3). Results are described as mean ± SD. *p<0.05, **p<0.01, ***p<0.001, ****p<0.0001, one-way ANOVA. Individual P value were listed in supporting information table S8.

**
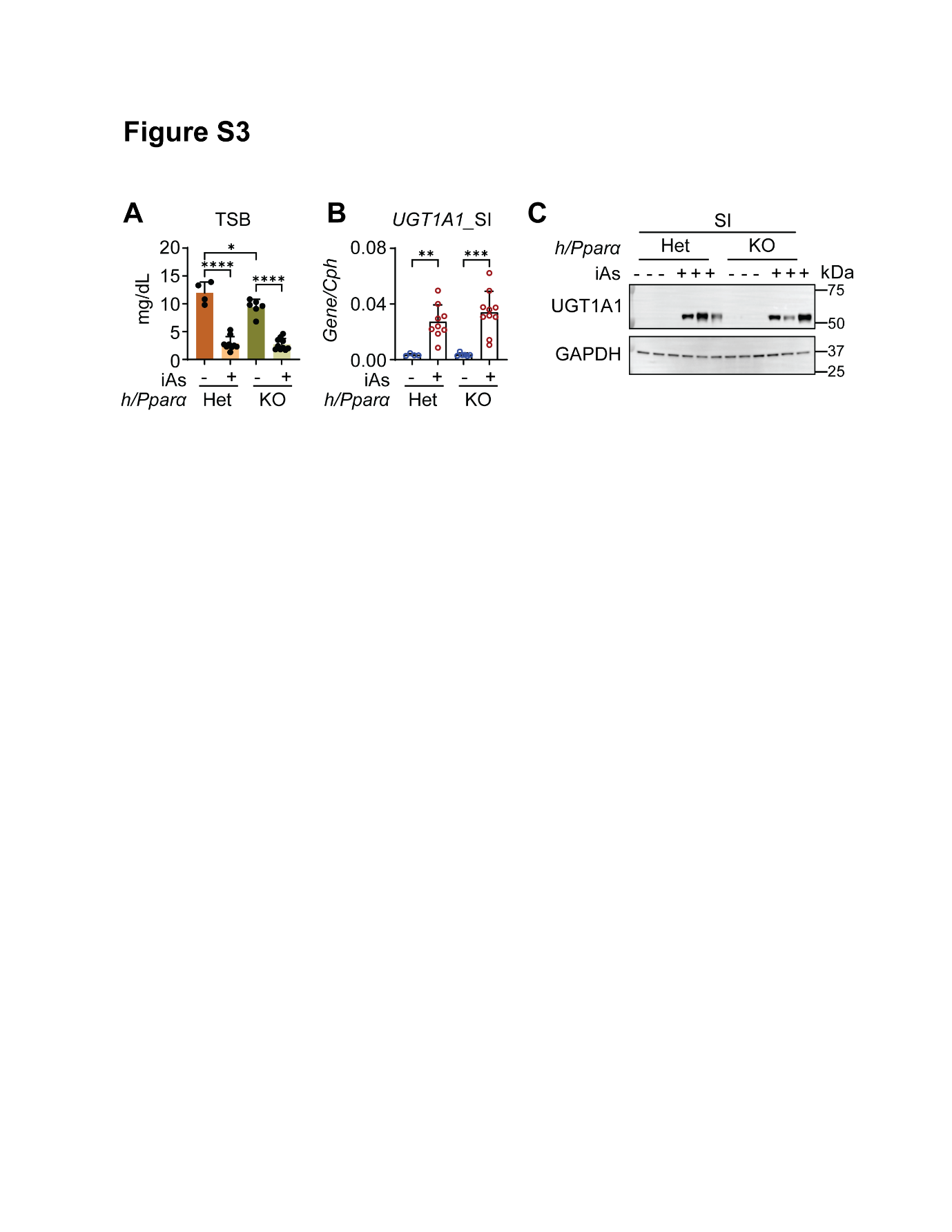
**

**Supporting Figure 3. iAs-mediated UGT1A1 induction is independent from PPARα**. hUGT1/Pparα^+/-^ and hUGT1/Pparα^-/-^ 12-day-old neonates were orally treated with iAs at 10 mg/kg for 48 h and small intestines collected. (**A**) TSB levels (n=4, 9, 6, 10). (**B**) RT-qPCR analysis of *UGT1A1* gene expression (n=4, 9, 6, 10). (**C**) Western blot analysis of UGT1A1 in the small intestine. Results are described as mean ± SD, *p<0.05, **p<0.01, ***p<0.001, ****p<0.0001, one-way ANOVA. Individual P value were listed in supporting information table S9.

**
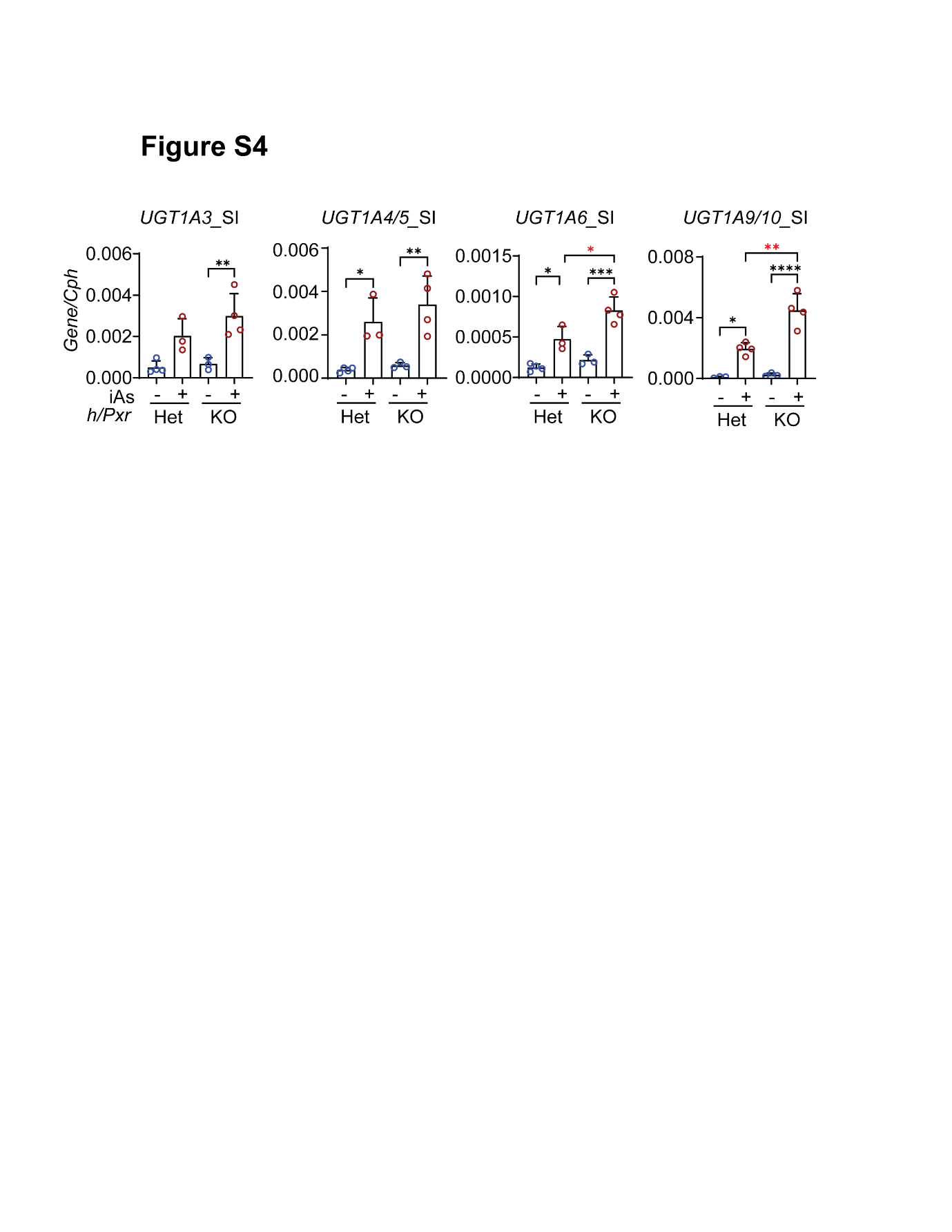
**

**Supporting Figure 4.** **The impact of PXR on expression of the *UGT1A* genes in small intestine**. Control and *hUGT1/Pxr^-/-^* 12-day-old neonates were orally treated with 10 mg/kg iAs. Small intestines were collected at 48h. RT-qPCR analysis of the different *UGT1A* genes (n=4, 3, 3, 4). Mean ± SD, *p<0.05, **p<0.01, ***p<0.001, ****p<0.0001, one-way ANOVA. Individual P value were listed in supporting information table S10.

**Supporting Table 1.** Primers used for real-time PCR analysis of selected genes

| **Gene** | **Forward** | **Reverse** |
| --- | --- | --- |
| *Akp3* | CTGGAGCCCTACACCGACT | AGGCTTCTGGCGCTGTTAT |
| *Cyp2b10* | TGCAGATGGACAGAGGAGG | CACACAGCATAACCACAGGC |
| *Cyp3a11* | TTCTGTCTTCACAAACCGGC | GGGGGACAGCAAAGCTCTAT |
| *Cyp4a10* | GATGGACGCTCTTTACCCAA | AAGGGTCAAACACCTCTGGA |
| *Hmox1* | GCAGGTGATGCTGACAGAGGA | GGGGGCCAGTATTGCATTTAC |
| *Lrp2* | CCAGGATTCTGGTGATGAGG | CGGGAACTCCATCACAAACT |
| *Nox4* | TCTGGAAAACCTTCCTGCTG | CCGGCACATAGGTAAAAGGA |
| *Nqo-1* | GGTGATATTTCAGTTCCCATTGC | GCAGGATGCCACTCTGAATC |
| *Sis* | ACCCCTAGTCCTGGAAGGTG | CACATTTTGCCTTTGTTGGATGC |
| *UGT1A1* | AACAAGGAGCTCATGGCCTCC | GTTCGCAAGATTCGATGGTCG |
| *UGT1A3* | TCAACTGTGCCAACAGGAAG | CTGAGACCATTGATCCCAAAG |
| *UGT1A4/5* | CAACGGGAAGCCACTATCTC | TGAGACCATTGATCCCAAAGA |
| *UGT1A6* | AATTTCCTAAAGGCCGGTCA | ACCACAATTCCATGTTCTCCA |
| *UGT1A7* | TGTCATCAGGGAAAGCCAGT | TGAGACCATTGATCCCAAAGA |
| *UGT1A9/10* | TGATGCCCAACATGATCTTC | CCACAATTCCATGTTCTCCA |

**Supporting Table 2.** Data analysis details for **Figure 1**.

| **Figure 1** | **Group** | **Analysis** | **P value** | **P value summary** |
| --- | --- | --- | --- | --- |
| *Fig 1A* | 24 vs.0 | Unpaired t test | <0.0001 | **** |
| *Fig 1A* | 48 vs.0 | Unpaired t test | <0.0001 | **** |
| *Fig 1B* | 24 vs.0 (SI) | Unpaired t test | 0.0176 | * |
| *Fig 1E*(*Sis*) | 2 vs.0 | Unpaired t test | <0.0001 | **** |
| *Fig 1E*(*Sis*) | 5 vs.0 | Unpaired t test | <0.0001 | **** |
| *Fig 1E*(*Sis*) | 10 vs.0 | Unpaired t test | <0.0001 | **** |
| *Fig 1E* (*Krt20*) | 5 vs.0 | Unpaired t test | 0.0002 | *** |
| *Fig 1E* (*Krt20*) | 10 vs.0 | Unpaired t test | 0.0076 | ** |
| *Fig 1F (Nox4)* | 10 vs.0 | Unpaired t test | <0.0001 | **** |
| *Fig 1F (Lrp2)* | 5 vs.0 | Unpaired t test | 0.0109 | * |
| *Fig 1F (Lrp2)* | 10 vs.0 | Unpaired t test | 0.0004 | *** |
| *Fig 1H (liver)* | iAs vs.C | Unpaired t test | 0.0015 | ** |

**Supporting Table 3.** Data analysis details for **Figure 2**.

| **Figure 2** | **Group** | **Analysis** | **P value** | **P value summary** |
| --- | --- | --- | --- | --- |
| *Fig 2B (Hmox-1)* | 4 vs.0 | Unpaired t test | 0.0146 | * |
| *Fig 2B (Hmox-1)* | 4 vs.0 | Unpaired t test | 0.0004 | *** |
| *Fig 2C (Nqo-1)* | 4 vs.0 | Unpaired t test | 0.0053 | ** |
| *Fig 2C (Nqo-1)* | 4 vs.0 (SI) | Unpaired t test | <0.0001 | **** |
| *Fig 2D (Gsta1)* | 4 vs.0 (liver) | Unpaired t test | 0.0203 | * |
| *Fig 2D (Gsta1)* | 24 vs.0 (liver) | Unpaired t test | <0.0001 | **** |
| *Fig 2D (Gsta1)* | 4 vs.0 (SI) | Unpaired t test | 0.0003 | *** |
| *Fig 2D (Gsta1)* | 24 vs.0 (SI) | Unpaired t test | 0.0017 | ** |
| *Fig 2D (Gsta1)* | 24 (SI) vs.24(liver) | Unpaired t test | 0.0228 | * |
| *Fig 2F (Cyp2b10)* | 48 vs.0 (liver) | Unpaired t test | 0.0409 | * |
| *Fig 2F (Cyp2b10)* | 48 vs.0 (SI) | Unpaired t test | 0.0223 | * |
| *Fig 2F (Cyp2b10)* | 48 (SI) vs.48(liver) | Unpaired t test | 0.0067 | ** |
| *Fig 2G (Cyp3a11)* | 24 vs.0 (liver) | Unpaired t test | 0.0319 | * |
| *Fig 2G (Cyp3a11)* | 24 vs.0 (SI) | Unpaired t test | 0.0047 | ** |
| *Fig 2H (Cyp4a10)* | 4 vs.0 (SI) | Unpaired t test | 0.0010 | *** |
| *Fig 2H (Cyp4a10)* | 24 vs.0 (SI) | Unpaired t test | <0.0001 | **** |

**Supporting Table 4.** Data analysis details for **Figure 3**.

| **Figure 3** | **Group** | **Analysis** | **P value** | **P value summary** |
| --- | --- | --- | --- | --- |
| *Fig 3A (Nqo-1)* | C vs. iAs (A-B) | One-way ANOVA | 0.0001 | *** |
| *Fig 3A (Nqo-1)* | iAs vs. iAs (B-D) | One-way ANOVA | 0.0006 | *** |
| *Fig 3A (Gsta1)* | C vs. iAs (A-B) | One-way ANOVA | <0.0001 | **** |
| *Fig 3A (Gsta1)* | C vs. iAs (C-D) | One-way ANOVA | 0.0008 | *** |
| *Fig 3A (Gsta1)* | iAs vs. iAs (B-D) | One-way ANOVA | <0.0001 | **** |
| *Fig 3B (Nqo-1)* | C vs. iAs (A-B) | One-way ANOVA | <0.0001 | **** |
| *Fig 3B (Nqo-1)* | iAs vs. iAs (B-D) | One-way ANOVA | <0.0001 | **** |
| *Fig 3B (Gsta1)* | C vs. iAs (A-B) | One-way ANOVA | <0.0001 | **** |
| *Fig 3B (Gsta1)* | iAs vs. iAs (B-D) | One-way ANOVA | <0.0001 | **** |
| *Fig 3D (UGT1A1)* | C vs. iAs (A-B) | One-way ANOVA | <0.0001 | **** |
| *Fig 3D (UGT1A1)* | C vs. iAs (C-D) | One-way ANOVA | 0.0199 | * |
| *Fig 3D (UGT1A1)* | iAs vs. iAs (B-D) | One-way ANOVA | 0.0148 | * |
| *Fig 3F (TSB)* | C vs. iAs (A-B) | One-way ANOVA | 0.0003 | *** |
| *Fig 3F (TSB)* | iAs vs. iAs (B-D) | One-way ANOVA | 0.0184 | * |
| *Fig 3G (Sis)* | C vs. iAs (A-B) | One-way ANOVA | <0.0001 | **** |
| *Fig 3G (Sis)* | C vs. iAs (C-D) | One-way ANOVA | 0.0207 | * |
| *Fig 3G (Sis)* | iAs vs. iAs (B-D) | One-way ANOVA | 0.0082 | ** |
| *Fig 3G (Krt20)* | C vs. iAs (A-B) | One-way ANOVA | 0.0440 | * |
| *Fig 3G (Krt20)* | C vs. iAs (A-C) | One-way ANOVA | 0.0237 | * |
| *Fig 3G (Krt20)* | iAs vs. iAs (B-D) | One-way ANOVA | 0.0102 | * |
| *Fig 3H (Cyp2b10)* | C vs. iAs (A-B) | One-way ANOVA | 0.0037 | ** |
| *Fig 3H (Cyp2b10)* | iAs vs. iAs (B-D) | One-way ANOVA | 0.0037 | ** |

**Supporting Table 5.** Data analysis details for **Figure 4**.

| **Figure 4** | **Group** | **Analysis** | **P value** | **P value summary** |
| --- | --- | --- | --- | --- |
| *Fig 4A (Cyp2b10)* | C vs. iAs (A-B) | One-way ANOVA | 0.0024 | ** |
| *Fig 4A (Cyp2b10)* | iAs vs. iAs (B-D) | One-way ANOVA | 0.0359 | * |
| *Fig4C (UGT1A1)* | C vs. iAs (A-B) | One-way ANOVA | 0.0004 | *** |
| *Fig4C (UGT1A1)* | C vs. iAs (C-D) | One-way ANOVA | 0.0018 | ** |
| *Fig 4D (TSB)* | C vs. iAs (A-B) | One-way ANOVA | <0.0001 | **** |
| *Fig 4D (TSB)* | iAs vs. iAs (B-D) | One-way ANOVA | <0.0001 | **** |
| *Fig 4E (Sis)* | C vs. iAs (A-B) | One-way ANOVA | 0.0039 | ** |
| *Fig 4E (Sis)* | C vs. iAs (C-D) | One-way ANOVA | 0.0013 | ** |
| *Fig 4F (Gsta1)* | C vs. iAs (A-B) | One-way ANOVA | 0.0041 | ** |
| *Fig 4F (Gsta1)* | C vs. iAs (C-D) | One-way ANOVA | 0.0020 | ** |

**Supporting Table 6.** Data analysis details for **Figure 5**.

| **Figure 5** | **Group** | **Analysis** | **P value** | **P value summary** |
| --- | --- | --- | --- | --- |
| *Fig 5A (TSB)* | C vs. iAs (A-B) | One-way ANOVA | <0.0001 | **** |
| *Fig 5A (TSB)* | C vs. iAs (C-D) | One-way ANOVA | 0.0012 | ** |
| *Fig 5A (TSB)* | iAs vs. iAs (A-C) | One-way ANOVA | <0.0001 | **** |
| *Fig 5B (UGT1A1)* | C vs. iAs (A-B) | One-way ANOVA | 0.0282 | * |
| *Fig 5B (UGT1A1)* | C vs. iAs (C-D) | One-way ANOVA | 0.0002 | *** |
| *Fig 5B (UGT1A1)* | iAs vs. iAs (B-D) | One-way ANOVA | 0.0402 | * |
| *Fig5D (UGT1A1)* | C vs. iAs (A-B) | One-way ANOVA | <0.0001 | **** |
| *Fig5D (UGT1A1)* | C vs. iAs (C-D) | One-way ANOVA | <0.0001 | **** |
| *Fig5D (UGT1A1)* | C vs. C (A-C) | One-way ANOVA | 0.0072 | ** |
| *Fig5D (UGT1A1)* | iAs vs. iAs (B-D) | One-way ANOVA | 0.0003 | *** |
| *Fig 5E (UGT1A1)* | iAs vs. iAs (B-D) | One-way ANOVA | 0.0353 | * |
| *Fig 5E (UGT1A1)* | C vs. iAs (C-D) | One-way ANOVA | 0.0360 | * |
| *Fig 5G (UGT1A1)* | iAs vs. iAs (B-D) | One-way ANOVA | 0.0008 | *** |
| *Fig 5G (UGT1A1)* | C vs. iAs (C-D) | One-way ANOVA | 0.0005 | *** |

**Supporting Table 7.** Data analysis details for supporting information **Figure S1**.

| **Figure S1** | **Group** | **Analysis** | **P value** | **P value summary** |
| --- | --- | --- | --- | --- |
| Fig S1A | 5 vs.0 | Unpaired t test | 0.0096 | ** |
| Fig S1A | 10 vs.0 | Unpaired t test | 0.0087 | ** |
| Fig S1B | 10 vs.0 | Unpaired t test | 0.0009 | *** |
| Fig S1C *(UGT1A3)* | 24 vs.0 | Unpaired t test | 0.0132 | * |
| Fig S1C *(UGT1A4/5)* | 24 vs.0 | Unpaired t test | 0.0117 | * |
| Fig S1C *(UGT1A6)* | 24 vs.0 | Unpaired t test | 0.0246 | * |
| Fig S1C *(UGT1A9)* | 24 vs.0 | Unpaired t test | 0.0365 | * |

**Supporting Table 8.** Data analysis details for supporting information **Figure S2**.

| **Figure S2** | **Group** | **Analysis** | **P value** | **P value summary** |
| --- | --- | --- | --- | --- |
| Fig S2A *(UGT1A3)* | C vs. iAs (A-B) | One-way ANOVA | <0.0001 | **** |
| Fig S2A *(UGT1A3)* | C vs. iAs (C-D) | One-way ANOVA | 0.0006 | *** |
| Fig S2A *(UGT1A3)* | iAs vs. iAs (B-D) | One-way ANOVA | <0.0001 | **** |
| Fig S2A *(UGT1A4/5)* | C vs. iAs (A-B) | One-way ANOVA | <0.0001 | **** |
| Fig S2A *(UGT1A4/5)* | iAs vs. iAs (B-D) | One-way ANOVA | <0.0001 | **** |
| Fig S2A *(UGT1A6)* | C vs. iAs (A-B) | One-way ANOVA | <0.0001 | **** |
| Fig S2A *(UGT1A6)* | iAs vs. iAs (B-D) | One-way ANOVA | <0.0001 | **** |
| Fig S2A *(UGT1A9/10)* | C vs. iAs (A-B) | One-way ANOVA | <0.0001 | **** |
| Fig S2A *(UGT1A9/10)* | iAs vs. iAs (B-D) | One-way ANOVA | <0.0001 | **** |
| Fig S2B *(Cyp4a10)* | C vs. iAs (A-B) | One-way ANOVA | 0.0059 | ** |
| Fig S2B *(Cyp4a10)* | C vs. iAs (C-D) | One-way ANOVA | 0.0001 | *** |
| Fig S2B *(Cyp4a10)* | C vs. C (A-C) | One-way ANOVA | 0.0340 | * |
| Fig S2B *(Cyp3a11)* | C vs. iAs (A-B) | One-way ANOVA | <0.0001 | **** |
| Fig S2B *(Cyp3a11)* | C vs. iAs (C-D) | One-way ANOVA | <0.0001 | **** |

**Supporting Table 9.** Data analysis details for supporting information **Figure S3**.

| **Figure S3** | **Group** | **Analysis** | **P value** | **P value summary** |
| --- | --- | --- | --- | --- |
| Fig S3A (TSB) | C vs. iAs (A-B) | One-way ANOVA | <0.0001 | **** |
| Fig S3A (TSB) | C vs. iAs (C-D) | One-way ANOVA | <0.0001 | **** |
| Fig S3A (TSB) | C vs. C (A-C) | One-way ANOVA | 0.0293 | * |
| Fig S3B *(UGT1A1)* | C vs. iAs (A-B) | One-way ANOVA | 0.0093 | ** |
| Fig S3B *(UGT1A1)* | C vs. iAs (C-D) | One-way ANOVA | 0.0001 | *** |

**Supporting Table 10.** Data analysis details for supporting information **Figure S4**.

| **Figure S4** | **Group** | **Analysis** | **P value** | **P value summary** |
| --- | --- | --- | --- | --- |
| Fig S4 *(UGT1A3)* | C vs. iAs (C-D) | One-way ANOVA | 0.0096 | ** |
| Fig S4 *(UGT1A4/5)* | C vs. iAs (A-B) | One-way ANOVA | 0.0327 | * |
| Fig S4 *(UGT1A4/5)* | C vs. iAs (C-D) | One-way ANOVA | 0.0082 | ** |
| Fig S4 *(UGT1A6)* | C vs. iAs (A-B) | One-way ANOVA | 0.0144 | * |
| Fig S4 *(UGT1A6)* | C vs. iAs (C-D) | One-way ANOVA | 0.0003 | *** |
| Fig S4 *(UGT1A6)* | iAs vs. iAs (B-D) | One-way ANOVA | 0.0145 | * |
| Fig S4 *(UGT1A9/10)* | C vs. iAs (A-B) | One-way ANOVA | 0.0158 | * |
| Fig S4 *(UGT1A9/10)* | iAs vs. iAs (C-D) | One-way ANOVA | <0.0001 | **** |
| Fig S4 *(UGT1A9/10)* | iAs vs. iAs (B-D) | One-way ANOVA | 0.0012 | ** |
